# Dynamic calibration of agent-based models using data assimilation

**DOI:** 10.1098/rsos.150703

**Published:** 2016-04-13

**Authors:** Jonathan A. Ward, Andrew J. Evans, Nicolas S. Malleson

**Affiliations:** 1 School of Mathematics, University of Leeds, Leeds LS2 9JT, UK; 2 School of Geography, University of Leeds, Leeds LS2 9JT, UK

**Keywords:** agent-based models, data assimilation, complex systems

## Abstract

A widespread approach to investigating the dynamical behaviour of complex social systems is via agent-based models (ABMs). In this paper, we describe how such models can be dynamically calibrated using the ensemble Kalman filter (EnKF), a standard method of data assimilation. Our goal is twofold. First, we want to present the EnKF in a simple setting for the benefit of ABM practitioners who are unfamiliar with it. Second, we want to illustrate to data assimilation experts the value of using such methods in the context of ABMs of complex social systems and the new challenges these types of model present. We work towards these goals within the context of a simple question of practical value: how many people are there in Leeds (or any other major city) right now? We build a hierarchy of exemplar models that we use to demonstrate how to apply the EnKF and calibrate these using open data of footfall counts in Leeds.

## 1. Introduction

Agent-based models (ABMs) are characterized by a set of rules, typically compiled together in a computer program, which describe the evolution of the model in time from an initial condition. The models are distinguished from others by representing individual actors in the modelled system *as* individuals, with their own behaviours and histories, often embedded with their own positions within a data environment. Such ‘agents’ may enact behaviour based on the same, or different, parameters and the rules they work to may vary from mathematical equations to adaptable artificial intelligence routines.

While agent-based systems can be used for a wide variety of purposes, including systems control, Web-search and robotics [1], ABMs are commonly implemented in science to understand social and environmental systems, either as simple *in silico* thought experiments or, increasingly, as detailed models of the real world [2]. In the latter case, the aim is not always just to understand the system studied, but also to predict it; early examples include eco-management [3], transport [4] and petrol prices [5]. One motivation for using properly calibrated ABMs to make predictions is that they can provide detailed information about the microscopic system state, something that could not be inferred from standard time series or statistical methods. At the moment, however, such predictive ABMs are generally calibrated once, using historical data to adjust their more flexible parameters such that the model predicts present and past conditions well. The models are then allowed to roll forward in time, independent of the world, to make a prediction. As the systems modelled are usually complex, it is likely that over longer time periods such models diverge from realistic estimates. Even over shorter time periods there is little done to check model performance, let alone constrain it.

This picture has largely resulted from the low data availability in the fields using ABMs. It has been rare that sufficient data have been available for both calibration and model initialization, let alone validation and what data there have been available have been collected at only a few time periods because of the associated expense. However, the rise of Web-based services, cheap field sensors and individual-level data collection and collation has recently led to a much greater availability of individual-level and environmental data, much of it streamed over near-continuous time. Such data offer the opportunity for models that use dynamic data assimilation (DDA) to constrain their continued predictive evolution against the real world [6]. In particular, there is a significant opportunity to draw from fields, such as meteorology, which have experience in error suppression during the predictive modelling of complex systems. Nevertheless, such attempts have been very limited. Areas where DDA could be applied to sociological and ecological models have typically been restricted to aggregated partial differential equation models, e.g. in the context of traffic flow [7,8] and urban crime [9], or for probabilistic and compartmental models of epidemic spreading [10–12]. Areas such as these are open to an ABM approach.

In this paper, we attempt to use DDA with an ABM as the fundamental framework. We focus on using the ensemble Kalman filter (EnKF), as ABMs are often highly nonlinear and can be computationally intensive. The EnKF is also relatively easy to implement, and thus seems a natural starting point for DDA with ABMs. In §3, we describe how the EnKF can be applied generically to ABMs and illustrate this in §4 using a simple example. As a more involved case study using real data, described in §6, we take the problem of estimating the number of people on a street in a major UK city, namely Leeds. In the case here, we formulate a behavioural model in §7 that captures the arrivals and departures of agents within a specific area in the city centre. In this area, cameras record hourly footfall counts and these data have been made available by the Leeds Data Mill [13]. In §8, we use these data to dynamically calibrate our model and describe the issues faced and our approach to overcome them.

## 2. Agent-based model formalism

Data assimilation methods, such as the EnKF, can be applied to generic dynamical models. Here we describe how ABMs fit into this framework. As noted earlier, ABMs encode in a computer program a set of behavioural rules that describe the evolution of the agents in the system. At any given instant, the state of the ABM is represented by many variables stored on the computer relating to, for example, the state of each agent and their environment. Such variables may include input and output data, as well as internal parameters and variable values. They may be real numbers, integers or categorical variables, but ultimately these can all be collected together in a single vector variable that describes the state of the model at a given time. In this paper, we will assume that this state vector has a fixed size. This does not exclude models where, for example, the number of agents changes in time. In reality, there will be an upper limit on the number of agents, dictated by the computer memory available, and the state vector would reflect this maximum possible size. Variables that are not in use can be set to zero allowing the state vector to be treated as sparse and thus passed efficiently between routines.

We now set up a precise description of the types of ABMs that we are interested in. If the state of the system has a fixed size then all possible states of the system belong to some finite state space X, and we will assume for the models of interest that this corresponds to the *n*-dimensional vector space of real numbers, i.e. $X:=R^{n}$. The state of the ABM evolves in some time set T, which could be continuous or discrete. However, in this paper we will be interested in the state of the model at particular times *t*_0_,*t*_1_,…,*t*_*T*_, with *t*_*k*_<*t*_*k*+1_, when we receive observations, and so we will focus on discrete times, i.e. $T:=\{t_{0},t_{1},\ldots ,t_{T}\}$. Even if the underlying model runs in continuous time (as ours will), we can still record the model state at the discrete observation times *t*_*k*_. We denote the state of the ABM at time *t*_*k*_ by $x_{k}\in X$ for *k*=0,…,*T*.

From a mathematical perspective, ABMs are typically stochastic processes: the decisions made by agents are dependent on draws from a random number generator. In this paper, we will focus on ABMs that are Markovian. While some ABMs track agent histories and use these data to determine the future evolution of the model, these can be recast as Markov processes by expanding the state vector to include the agent histories. Markov processes are typically defined in terms of their transition matrix *P*(*x*_*k*+1_ | *x*_*k*_); however, this is not the case for the ABMs we are interested in. Instead, we are only able to sample this distribution via simulation. Also, in most of the literature on data assimilation of stochastic processes, the dynamical model is written as a stochastic differential or difference equation, e.g.

$$x_{k+1}=F(x_{k})+\upsilon_{k},$$

where F:X→X is a deterministic map and $\upsilon_{k}\in X$ is a random variable with known probability distribution. While it may be possible in theory to write the ABMs of interest to social scientists in this form, in practice, this is typically not tractable without approximation.

Instead, we use the following formalism. We assume that our ABM is defined by a fixed set of rules and stochasticity results from a finite set of conditions within these rules that are dependent on random numbers encoded in a random variable $\xi_{k}\in R^{q}$. Thus, the programmatic implementation of our ABM can be represented by a time-dependent *deterministic* map between states $M_{k}:X\times R^{q}\to X$ that takes the stochastic variable *ξ*_*k*_ as an input, specifically^1^

2.1 $$X_{k+1}=M_{k}(x_{k};\xi_{k}),$$

where *X*_*k*+1_ is a random variable corresponding to the state of the system at time *t*_*k*+1_. Formally, *ξ*_*k*_ is associated with a probability space and so we denote a particular realization by *ξ*_*k*_(*ω*), where *ω*∈*Ω* is an element of the sample space *Ω* of *ξ*_*k*_. As *ξ*_*k*_ entirely characterizes the stochasticity of our simulation, we similarly denote a particular realization of the ABM by the lower case variable *x*_*k*_:=*X*_*k*_(*ω*).

## 3. The ensemble Kalman filter

With our formulation of ABMs in place, we now describe the EnKF for the purpose of state estimation. This follows closely the description in ch. 30 of Lewis *et al.* [14], to which we direct the reader for further details. In §4, we illustrate how the same basic procedure can be extended to sequential parameter estimation.

The EnKF is an iterative method that uses two basic steps, roughly described as follows:

(i) In the *forecast step*, an ensemble of estimated model states, ${\hat{x}}_{k}(i)$ at time *t*_*k*_ for *i*=1,…,*N*, are evolved forward independently until the next observation time *t*_*k*+1_, yielding an ensemble of *forecasts* *x*^*f*^_*k*+1_(*i*). The mean of the ensemble forecast states, *x*^*f*^_*k*+1_, provides an estimate of the true state at the future time *t*_*k*+1_ and the covariance of the ensemble forecast states, $P_{k+1}^{f}$, provides a measure of its uncertainty.
(ii) In the *data assimilation step*, after the observation *z*_*k*+1_ is received, the ensemble forecasts are updated in the light of the new data. The collection of updated variables, ${\hat{x}}_{k+1}(i)$, is called the ensemble *analysis*. The mean of the ensemble analysis states, ${\hat{x}}_{k+1}$, provides a best guess of the system state and the covariance of the ensemble analysis states, ${\hat{P}}_{k+1}$, provides an estimate of its uncertainty.

This process can then be repeated iteratively. It is initialized with knowledge or an estimation of the initial system state and its covariance.

We now describe the EnKF algorithm in more detail. To produce the forecasts, the ensemble of model states ${\hat{x}}_{k}(i)\in R^{n}$ at time *t*_*k*_ are used as initial conditions in the dynamical model and evolved forward to the next observation time *t*_*k*+1_. Specifically, our ABM produces forecast realizations $x_{k+1}^{f}(i):=X_{k+1}^{f}(\omega )\in R^{n}$ of the random variable

3.1 $$X_{k+1}^{f}=M_{k}({\hat{x}}_{k}(i);\xi_{k}).$$

The average of the forecast ensemble

$$x_{k+1}^{f}=\frac{1}{N}\sum_{i=1}^{N}x_{k+1}^{f}(i)$$

provides an estimate of the forecasted system state and we can use the ensemble forecast error

$$e_{k+1}^{f}(i)=x_{k+1}^{f}(i)-x_{k+1}^{f}$$

to determine the forecast covariance

$$P_{k+1}^{f}=\frac{1}{N-1}\sum_{i=1}^{N}e_{k+1}^{f}(i)[e_{k+1}^{f}{\left(i)\right]}^{T}.$$

This quantifies the uncertainty in our forecast estimate of the system state.

In the data assimilation step, we assume that the observation $z_{k}\in R^{m}$ is a linear function of the true state of the system plus additive Gaussian noise, i.e.

3.2 $$z_{k}=H_{k}x_{k}+\nu_{k},$$

where $\nu_{k}∼N(0,R_{k})$ and the matrix $H\in R^{m\times n}$ is called the forward model. The covariance matrix $R_{k}\in R^{m\times m}$ represents our uncertainty in the observation. Following the observation *z*_*k*+1_ at time *t*_*k*+1_, we produce an ensemble of ‘virtual observations’, given by

$$z_{k+1}(i)=z_{k+1}+\nu_{k+1}(i),$$

where $\nu_{k+1}(i)∼N(0,R_{k+1})$. The use of virtual observations provides a better estimate of the posterior covariance [14]. These are then used to adjust the states of the ensemble and produce the ensemble analysis

$${\hat{x}}_{k+1}(i)=x_{k+1}^{f}(i)+K[z_{k+1}(i)-H_{k+1}x_{k+1}^{f}(i)],$$

where $K\in R^{n\times m}$ is the ‘Kalman gain matrix’. This shifts the forecast by an amount relative to the difference between the predicted observations $H_{k+1}x_{k+1}^{f}(i)$ and the virtual observations *z*_*k*+1_(*i*). The Kalman gain matrix, given by

$$K=P_{k+1}^{f}H_{k+1}^{T}{\left[H_{k+1}P_{k+1}^{f}H_{k+1}^{T}+R_{k+1}\right]}^{-1},$$

is chosen to reduce the analysis variance and for the case of a linear model it provides the minimum variance as N→∞.

Finally, we average the ensemble analysis

$${\hat{x}}_{k+1}=\frac{1}{N}\sum_{i=1}^{N}{\hat{x}}_{k+1}(i)$$

to approximate the system state at time *t*_*k*+1_. Denoting the error of the *i*th ensemble member by

$${\hat{e}}_{k+1}(i)={\hat{x}}_{k+1}(i)-{\hat{x}}_{k+1},$$

the ensemble analysis covariance is given by

$${\hat{P}}_{k+1}=\frac{1}{N-1}\sum_{i=1}^{N}{\hat{e}}_{k+1}(i){\left[{\hat{e}}_{k+1}(i)\right]}^{T}.$$

The mean of the ensemble analysis provides an estimate of the true state in the light of the observed data and the analysis covariance quantifies its uncertainty. This completes one step of the EnKF.

The EnKF update of the forecast state $x_{k+1}^{f}$ and covariance $P_{k+1}^{f}$ is a non-deterministic linear coupling. While the choice of this coupling is not unique, using the Kalman gain matrix K with virtual observations ensures that ${\hat{x}}_{k+1}$ and ${\hat{P}}_{k+1}$ are best unbiased linear estimates [15] and, in the special case of linear dynamics, the EnKF approaches the standard Kalman filter for large *N*. For more details, see ch. 30 of Lewis *et al.* [14].

## 4. A simple example

To demonstrate the EnKF at work, we consider a simple but relevant model of arrivals and departures of people from a given area, e.g. a shop, a district or a city. In this section, we describe this model and its dynamics, and in the next section, we apply the EnKF to it to make forecasts about the model state and its parameters. We imagine that the area of interest in the model is a ‘box’ that can hold any number of people. In addition, we suppose that people arrive in the box at random times but with a fixed average rate *α*, and each person departs at a different fixed average rate *β*. Thus, if *x*(*t*) is the number of people in the box at time *t*, then the average rate of departures is *βx*(*t*). We simulate this model using the Gillespie algorithm^2^ [16] from an initial condition *x*_0_:=*x*(0).

This model is sufficiently simple that we can determine explicit expressions for its time evolution. The master equation for the probability *p*_*n*_(*t*) that there are *n* people in the box at time *t* is

4.1 $${\dot{p}}_{n}=\alpha p_{n-1}+\beta (n+1)p_{n+1}-[\alpha +\beta n]p_{n}(t),$$

for *n*≥0. From this master equation, we can easily determine the evolution of the mean and variance in the number of people. Let $u(t)=\sum_{n=0}^{\infty }np_{n}(t)$ be the mean, then substitution into (4.1) yields a linear first-order ordinary differential equation

$$\dot{u}=\alpha -\beta u,$$

which has solution

4.2 $$u(t)=\gamma +(u_{0}-\gamma )e^{-\beta t},$$

where *γ*=*α*/*β*. We can also compute the variance in the number of people

$$v(t)=\sum_{n=0}^{\infty }{\left(n-u\right)}^{2}p_{n}(t),$$

which on substitution into (4.1) yields a linear inhomogeneous first-order ordinary differential equation

$$\dot{v}=\alpha +\beta u-2\beta v.$$

Using the solution for *u*(*t*), we find

4.3 $$v(t)=\gamma +(u_{0}-\gamma )e^{-\beta t}+[v_{0}-\gamma -(u_{0}-\gamma )]e^{-2\beta t}.$$

It is also possible to determine an explicit solution for the distribution *p*_*n*_(*t*) via generating functions.

**Figure 1.**
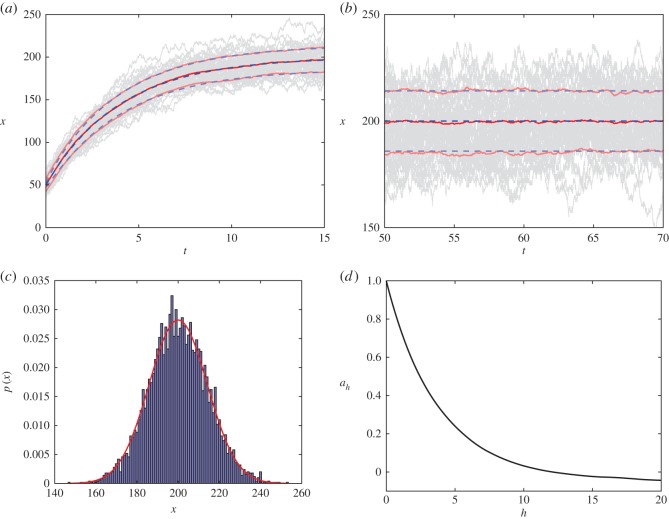
Illustration of the dynamics of the simple box model. (*a*,*b*) Time-series trajectories of stochastic simulation realizations (grey) and the corresponding averages in red. The mean predicted from theory is illustrated in dashed blue. (*c*) A histogram of the ensemble at *t*=60 and (*d*) the autocorrelation at different lags *h*. More details are included in the main text.

In figure 1, we illustrate results from 200 stochastic simulation realizations of our box model and compare them with the expressions derived from theory. The simulations used to produce the results depicted in figure 1*a*–*d* all correspond to parameter values *α*=50 and *β*=0.25 computed from initial conditions sampled from a Poisson distribution with mean ${\bar{x}}_{0}=5$. Note that 1/*β*=4 gives the average time spent in the box. Figure 1*a* illustrates the time evolution up to *t*=15. Individual realizations are plotted in light grey (20 chosen at random from the ensemble). The mean of the 200 realizations is plotted in red and the mean ± 1 s.d. in light red. The theoretical mean, given by (4.2), is plotted in dashed blue and the theoretical mean ± 1 s.d., as determined by the square root of (4.3), is plotted in dashed light blue. The dynamics are particularly simple—the state variable *x* evolves stochastically towards a steady state given by *α*/*β*, around which individual stochastic realizations fluctuate. The scale of these fluctuations around the steady state is $\sqrt{\alpha /\beta }$, illustrated in figure 1*b*, whose format is the same as figure 1*a* but depicts a later time interval. In figure 1*c*, we show a histogram of states *x*(*T*) with *T*=60 from 5000 realizations. The histogram bins are the integers and plotted in red is a Gaussian distribution with the theoretical mean and variance at *T*=60, which gives a good approximation to the distribution. In figure 1*d* , we illustrate the autocorrelation *a*_*h*_ for different time lags *h* computed from 500 realizations, simulated up to *t*=180 using 4500 equally spaced observations. The decay of the autocorrelation function is exponential and characterizes the time scale over which previous states are ‘forgotten’.

### 4.1. State estimation

Having described our box model and its dynamics, we now illustrate how the EnKF can be used with the box model to make predictions about noisy observations. Before applying the EnKF, we created synthetic observation data from a single realization of the model which we took as the ground truth state. Note that in this example the model used by the EnKF is the same as that used to generate the observation data, whereas with real data our model would only be an approximate description of the true data generating process. Furthermore, when applying the EnKF to real data we do not have knowledge of the true state, only what we get from (noisy) observations.

The synthetic ground truth data consisted of the number of people in the box *x*_*k*_ at times *t*_*k*_=*k* for *k*=0,1,…,100. In computing the ground truth data, we used the rate parameters *α*≈52.52, chosen at random from a normal distribution with mean 50 and variance 25, and *β*≈0.2580, chosen at random from a normal distribution with mean 0.25 and variance 10^−4^ (we use random samples because we will also use this synthetic data for sequential parameter estimation in the next subsection). The initial condition was also chosen at random from a rounded normal distribution with mean 200 and variance 200, and in this instance we drew *x*_0_=200. We produced observations *z*_*k*_ from the ground truth *x*_*k*_ by adding Gaussian noise with mean zero and variance *R*=16.

**Figure 2.**
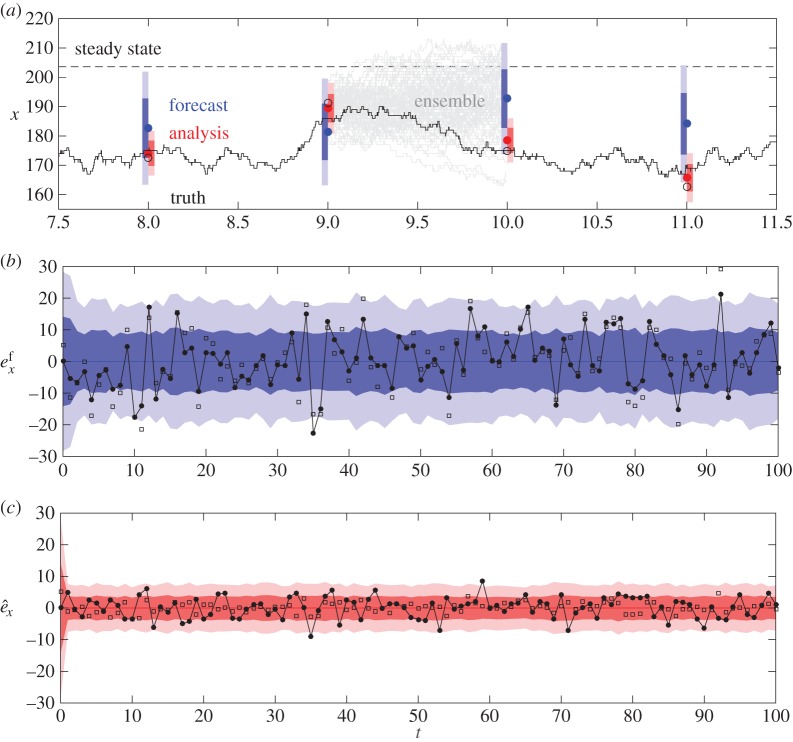
Illustration of the EnKF applied to the box model. (*a*) Synthetic ground truth data are represented by the solid black line, noisy observations are indicated by open circular black markers, the forecasts by blue markers and the analysis by red markers. Individual ensemble realizations are plotted in grey. (*b*) and (*c*) illustrate the forecast and analysis errors, respectively. More details are included in the main text.

For the EnKF, we initialized each of the *N*=100 ensemble simulations using a rounded normal distribution with mean 200 and variance 200, as we did for the ground truth data, and used the corresponding rate parameters *α*≈52.52 and *β*≈0.2580. Note that as the simulation directly produces the quantity we are observing, namely the number of people in the box, we have H=1. The EnKF produces estimates of the true state *x*_*k*_ at the observation times *t*_*k*_. The estimate before the observation is the forecast *x*^*f*^_*k*_, and after is the analysis ${\hat{x}}_{k}$. Figure 2*a* illustrates a snap shot of the EnKF at work. The ground truth synthetic data *x*_*k*_ is plotted in black and the theoretical mean, *u*=*α*/*β*≈203.5, is indicated by a dashed black line. Black markers correspond to the noisy observations *z*_*k*_, blue markers to the forecasts *x*^*f*^_*k*_ and red markers to the analysis following observations ${\hat{x}}_{k}$. The means ± 1(2) s.d. for the forecast are shaded in blue (light blue) and similarly for the analysis in red (light red). To illustrate the dynamics of the ensemble, we plot the individual realizations between observations at *t*=9 and *t*=10 in light grey. Note that the analysis uncertainty is always less than the forecast uncertainty. The ensemble averaged forecasts tend to move towards the steady state, so it is important that the observation data occur sufficiently frequently that the dynamics are not dominated by stochastic fluctuations.

In figure 2*b*, we illustrate the forecast errors. Specifically, we use the forecast *x*^*f*^_*k*_ as the baseline at zero, indicated by the blue line, and the error between the forecast and ground truth, $e_{k}^{f}=x_{k}-x_{k}^{f}$, in black with circular markers. We also include the difference between the forecast and observation, *z*_*k*_−*x*^*f*^_*k*_, indicated with square markers. This allows us to visualize the forecast variance *P*^*f*^_*k*_ clearly. The shaded dark blue region corresponds to $\pm \sqrt{P_{k}^{f}}$ and the shaded light blue region corresponds to $\pm 2\sqrt{P_{k}^{f}}$. The behaviour illustrated is typical—the forecast variance quickly decays to a value that then remains roughly constant. Similarly, in figure 2*c*, we illustrate the analysis errors ${\hat{e}}_{k}=x_{k}-{\hat{x}}_{k}$ plotted in black with circular markers using the analysis ${\hat{x}}_{k}$ as the baseline, plotted in red. The differences $z_{k}-{\hat{x}}_{k}$ are plotted using square markers. We use the same scale as figure 2*b* to illustrate that the analysis errors are much smaller than the forecast errors.

For the above case, we have forecast RMSE: 8.4557; analysis RMSE: 3.3648; observation RMSE: 3.8177 (i.e. the difference between the observations with measurement error and the synthetic ground truth). Note that the analysis RMSE is *better* than the observation RMSE. This illustrates a key point. Knowledge of the underlying process should allow us to make a better estimate of the true state than noisy observations.

Our simple box model has very simple dynamics, as illustrated in figure 1, but the initial conditions used in this example were deliberately chosen near to the steady state *α*/*β*. Thus, the ground truth simulation trajectory simply fluctuates randomly around this steady state. With this in mind, a very simple forecast would be the steady state calculated from theory. This gives an RMSE of 13.3465, worse than all of the RMSEs listed above. This highlights another feature of the EnKF. Even in the absence of dynamical behaviour, the EnKF allows us to take advantage of autocorrelations in order to approximate the true system state.

### 4.2. Sequential parameter estimation

In addition to state estimation, we can also use the EnKF to make predictions about unknown parameters [14,15] by including them in the EnKF state vector. This is known as sequential parameter estimation. From a practical perspective, this necessitates an interface script that takes the parameters and model state variable from the EnKF state vector and puts them into the correct format for the model evolution routine.

**Figure 3.**
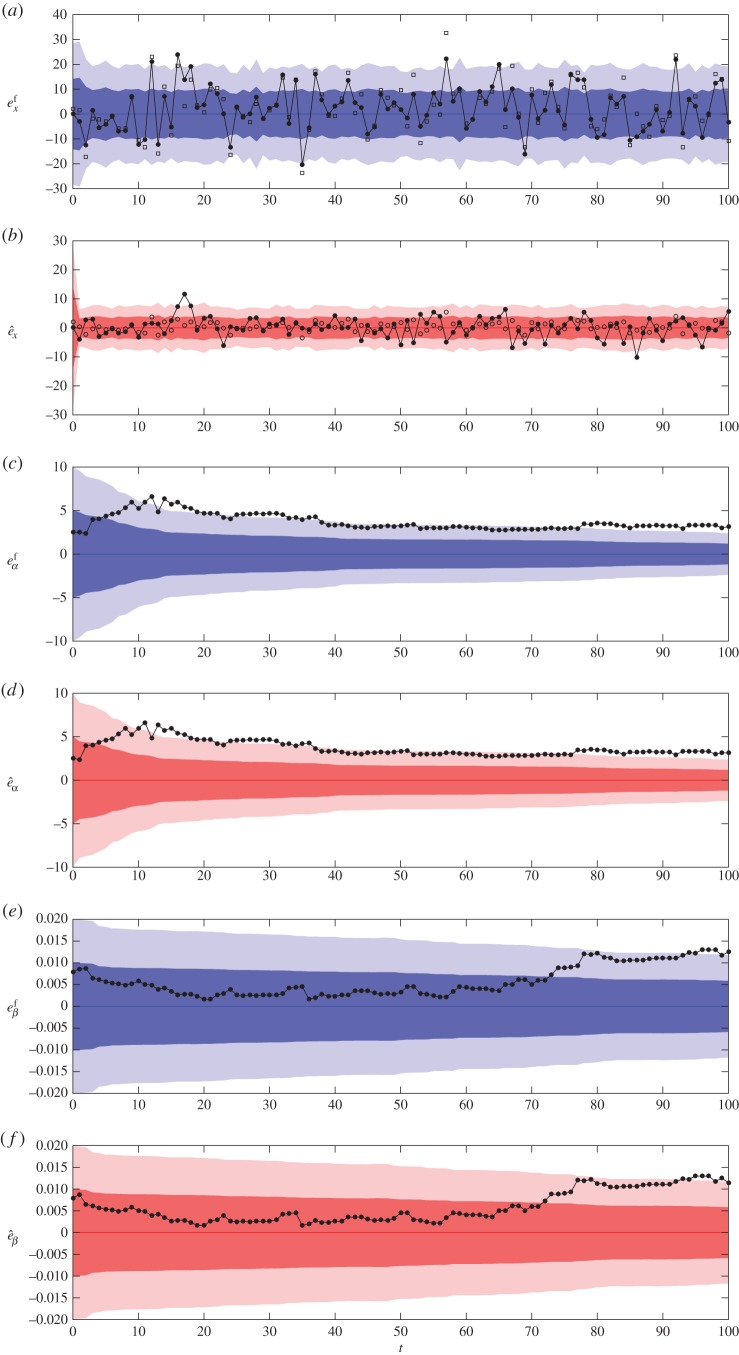
Forecast (blue) and analysis (red) errors for sequential parameter estimation of the simple box model. (*a*,*b*) The corresponding errors for *x*, (*c*,*d*) for *α* and (*e*,*f*) for *β*. More details are included in the main text.

In figure 3, we illustrate sequential parameter estimation applied to the simple box model using exactly the same ground truth data as used in §4a. In this case, we do not assume knowledge of the parameters *α* and *β*, and initialize the ensemble using the distributions described in §4a, namely normal distributions with means 50 and 0.25, and variances 25 and 10^−4^, respectively. Thus at each observation time, the EnKF produces a forecast of the parameters, denoted *α*^*f*^_*k*_ and *β*^*f*^, and an analysis, denoted by ${\hat{\alpha }}_{k}$ and ${\hat{\beta }}_{k}$, in addition to the forecast and analysis of the model state. In figure 3*a*,*b*, we plot the forecast and analysis errors, respectively, of the number of people in the box *x*. Note that the forecast errors are larger in this case than in §4a, but the analysis errors are roughly the same. We also illustrate the forecast and analysis errors for the arrival rate parameter *α* in figure 3*c*,*d*, respectively, and similarly for the departure rate parameter *β* in figure 3*e*,*f*, respectively. The errors,

$$\begin{array}{lll} e_{\alpha }^{f}(k)=\alpha -\alpha_{k}^{f}, & & e_{\beta }^{f}(k)=\beta -\beta_{k},\\ {\hat{e}}_{\alpha }(k)=\alpha -{\hat{\alpha }}_{k} & \text{and} & {\hat{e}}_{\beta }(k)=\beta -{\hat{\beta }}_{k}, \end{array}$$

are plotted in black. We use the *forecast* and *analysis* estimations of the parameters as the base line, indicated by blue and red lines, respectively. Note that the actual ground truth parameters are constant. In addition, changes in the estimates of the parameters in the EnKF are driven solely by their covariance with *x*_*k*_, the number of people in the box.

Figure 3*c*–*e* show that while the uncertainty around the estimated parameter values decreases in time, the estimates do not necessarily get closer to the true values. In fact, at the end of the simulation period the true values lie outside of the two standard deviation range around the estimated values. However, the *ratio* of the estimated parameters, which corresponds to the steady state, is 201.1 for the forecast and 200.3 for the analysis, the true value being 203.5. From §4, we know that it is this ratio that governs the stationary statistics and so in this sense the EnKF has found the correct parametric representation of the synthetic data. This observation shows that understanding the relationship between model parameters and model dynamics is important if we are to correctly interpret the results of sequential parameter estimation when using the EnKF.

For our sequential parameter estimation, we have forecast RMSE: 9.3456; analysis RMSE: 3.4938 and observation RMSE: 3.8682. While the forecast and analysis RMSEs are larger than for the state estimation case, the analysis RMSE is still smaller than the observation RMSE.

## 5. Case study background

We now proceed to a more involved case study in which we develop a model that can be used to estimate hourly footfall counts on a pedestrian high street. There has been long-standing interest in modelling pedestrian behaviour. Early approaches adapted models from fluid dynamics [17,18] and magnetism (often termed ‘social-force’) [19–21]. More recently, ABMs that incorporate individual-level advanced cognitive behaviour have been able to accurately simulate the dynamics of human crowds [22,23]. While models of detailed crowd dynamics are essential for some applications (such as evacuation [24] and public gatherings [25]), these models are not usually applied in areas where crowd density is low. In the application discussed here, only very rare events will cause the density of the crowd to reach a point that it changes individual shopper behaviour. Hence an advanced model that realistically represents individual trajectories adds unnecessary complexity.

There is also a rich literature from the field of retail geography that attempts to better understand what attracts people to certain shops and, ultimately, where they will choose to visit. Early efforts handled the problem with an Intervening Opportunities Model [26], in which a starting population entering a street is subdivided between shops approached from a particular direction, and with a given probability of attracting customers from the street. More recently, techniques such as spatial interaction models [27,28] have attempted to estimate the volumes of flows from origins (homes) to destinations (shops). However, these approaches largely model populations at the aggregate level—an approach that has led to difficulties with aspects such as modelling multi-purpose trips. Models might provide estimates of the number of pedestrians in a shopping district, but not their movements between shops on the high street. This limitation has been well known for some time [29,30].

To this end, a complementary literature has also grown around the concept of route choice and, in particular, what drives individuals to choose certain shops. For example, Borgers & Timmermans [31] attempt to model the behaviour of pedestrians in a ‘downtown’ area who are motivated by shopping, strolling or by other factors, using a multinomial logit model. The rules that govern the model are largely distance based; as individuals move farther away from their entry point they are gradually driven back to where they started. In a similar vein, Hoogendoorn & Bovy [32] present a model of pedestrians as *subjective utility maximizers*. However, although these examples (and others like them) have proved interesting, they also operate at an aggregate level and hence face difficulties incorporating individual heterogeneity. To avoid this drawback, microscopic/individual-level studies have also begun to emerge. One of the earliest agent-based pedestrian models, entitled STREETS [33], was able to create ‘plausible’ emergent patterns (although, as the authors admit, it was only loosely validated). Similarly, Asano *et al.* [34] implement a model with two complementary sub-processes: a *tactical* process that finds an optimal shortest path for an agent and an *operational* process that dynamically adapts the route to avoid other pedestrians, etc. The authors note that such models are valuable in maintaining a certain level of service in busy pedestrian areas such as ‘busy stations and event venues’ [35] (cited in [34]). As noted above, however, a detailed pedestrian route-choice model is unnecessary here. Directness and distance are the most common strategies for deciding on a route [32,36]. Therefore, as before, a detailed model of route choice adds unnecessary complexity.

## 6. Data and study area

This paper explores the potential offered by DDA in a particular study area: the high street *Briggate* in Leeds, UK. In order to monitor footfall in the city centre, Leeds City Council commissioned the installation of cameras at a number of locations around the city. The cameras track pedestrian movements and count the number of people who pass their location. These data are subsequently aggregated to hourly flow counts and released publicly as part of the Leeds City Centre Footfall dataset available on the Leeds Data Mill [13]. There are two cameras located on Briggate, one near the Headrow at the northern end of the street and another at the exit of Briggate near Boar Lane/Duncan Street. As discussed, the camera data include no estimate of total population or flow direction. Furthermore, Briggate is not a closed system. Pedestrians can enter or leave without passing the points covered by cameras.

The footfall count data consist of integer counts of people during each hour of the day. In this paper, we will restrict our attention to the Briggate camera. This dataset has counts every hour from 00:00 on Friday 20 July 2007 to 23:00 on Thursday 30 January 2014 (times are in 24 h), 2387 days in total. The minimum count is 0, of which there are 14 occurrences (6 of which occur on Thursday 31 July 2008 between 18:00 and 23:00), and the maximum count is 19 820, which occurs at 12:00 on Thursday 26 December 2013.

It proved infeasible to run our EnKF algorithm over the entire dataset, so we chose a six-week period between 00:00 on Saturday 5 May 2012 and 23:00 on Friday 16 June 2012 as a *testing* dataset. This period was chosen to enable us to simulate a reasonable period of time because the counts over this period stay relatively low. The following data analysis uses only data prior to that date, which we consider as *training* data for our model development.

**Figure 4.**
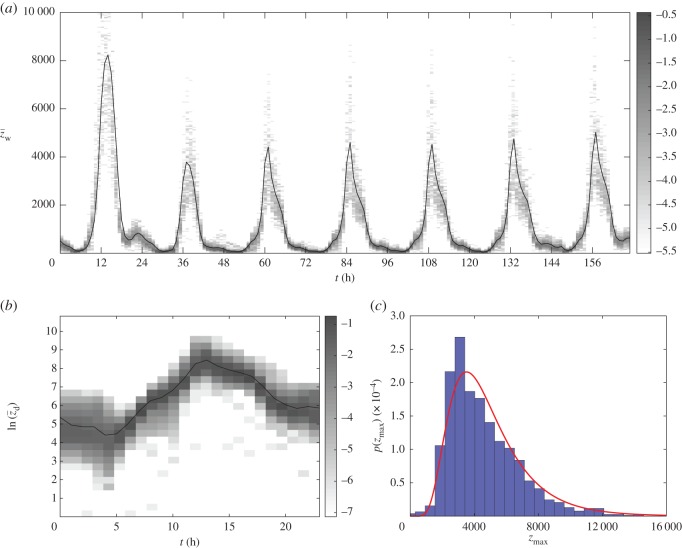
Statistics of Briggate footfall data. (*a*) The mean hourly counts from Saturday to Friday. (*b*) The mean hourly counts of just weekdays (Monday to Friday) together. (*c*) The distribution of peak daily counts and comparison with a lognormal distribution. More details are included in the main text.

In figure 4, we illustrate our key observations from the footfall data. The mean hourly counts across days of the week, ${\bar{z}}_{w}$, starting from Saturday, are plotted in black in figure 4*a*. In addition, the shading corresponds to the log of the probability that the observed count lies within the corresponding 50 count interval. For illustrative purposes, the vertical scale does not capture the full spread of values that occur. On average, Saturdays are the busiest days of the week, and Sundays are the least busy. As the profiles between different weekdays (Monday to Friday) are similar but Saturdays and Sundays are distinct, we decided to restrict our attention to predicting weekday behaviour. This meant that the next reading after 23:00 on Friday was 00:00 on Monday, but we believe the data are sufficiently noisy that this does not cause an issue. Combining weekdays into a single 24 h period, ${\bar{z}}_{d}$, in figure 4*b*, we plot the same type of plot as in figure 4*a* but scale the footfall counts logarithmically. This illustrates clearly that there are three distinct periods during the day when the growth or decay in the number of counts is approximately exponential.

In figure 4*c*, we also plot the distribution of peak weekday counts and compare it with a lognormal distribution with the corresponding exponential mean and variance. This shows that there is extremely high variability between daily peak counts. Moreover, the correlation between peak counts of consecutive days is only approximately 0.30. Thus, today being busy is not a good indication of whether tomorrow will be busy. We will see in §7 that this daily volatility of counts proves challenging for behavioural models.

## 7. WHIRS model

Our first attempt to come up with a relatively simple behavioural model with which we could forecast footfall counts was not successful, but provided some key insights. In this model, we considered the length of Briggate to be made up of discrete one-dimensional cells. Agents could arrive at various locations, corresponding to the true geometry of Briggate, and would proceed to ‘walk’ by hopping between neighbouring cells. As well as walking, agents could also enter and exit shops at specified rates before returning to the street and continuing to walk. Agents could leave the street at a fixed rate from certain designated places or could walk off either end of the street, removing them from the simulation. We assumed that the arrival rates varied piecewise linearly across the day. We included virtual cameras that observed the number of agents crossing certain points corresponding to the locations of the footfall cameras, within each hour of the day.

However, this naive modelling approach had two fundamental issues. First, given a realistic walking speed most agents could traverse the length of Briggate within an hour—Briggate is approximately 320 m long, average walking speed is around 5 kph and so it would take approximately 4 min to walk its length, excluding any time spent in shops. Consequently, the camera observations from our simulation were highly correlated with the arrival rates, and this persisted even when we included shopping behaviour. Thus, within the parameter values investigated, the behavioural components of this model (walking and shopping) had little effect on the virtual footfall counts recorded in simulations. We discovered this while trying to fit this model to the observation data because there was a weak dependence of the simulated observations on the behavioural parameters and so these were not constrained by the observation data. Thus, it would be erroneous to draw conclusions from the dynamics of the fitted model about real behaviour. But with no behavioural element, we may also use standard time series or statistical methods to forecast the footfall counts.

The second issue with this model is that, while the arrival rates may vary during the course of a day, following the analysis of our simple model in §4, the spread in the distribution of the peak number of arrivals (and consequently the number of camera counts) should go roughly like the square root of the peak number of arrivals. The average number of arrivals observed in the data is approximately 5000, so if our simulations replicate this we would observe a standard deviation of around 70, which corresponds to less than a 2% variation. However, as illustrated in §6, in the real data the standard deviation around the mean is much larger, around 2000, approximately 45% of the mean. Thus, the stochasticity of our first modelling approach woefully underestimates the true spread in the peak camera counts.

It seems likely that the underlying volatility of the peak camera counts may be due to external factors, such as the weather, public holidays and sporting or music events. A model that requires knowledge of such events is not well placed to make forecasts using the footfall data alone, as is our goal, as such events may themselves be unpredictable (the weather) or may require additional data (e.g. times and locations of sporting events). Our goal is to produce a plausible model that does not require such prior information.

To achieve this, we assume that knowledge of such events is shared by the agents in our system, and thus the number of people in the system on a given day can be used as a proxy for whether that day will attract more or less people. Thus, in a similar manner to epidemic models, we propose a ‘rich gets richer mechanism’ for the arrival of people into the city. In this first instance, we do not consider the geometry of the city centre and focus on the data collected by only the Briggate camera. We assume a large, but fixed, number of agents *N* in our model who form the pool of people who may walk past the footfall camera. Each of these agents is either a shopper or a worker. Shoppers can be in one of three states: *susceptible* to going shopping, denoted by S; *in town*, denoted by I; or *returned* home, denoted by R. Workers can be in one of two states: at *home*, denoted by H; or at *work*, denoted by W. Shoppers cannot become workers and vice versa, so the number of workers *N*_*w*_ and the number of shoppers *N*−*N*_*w*_ remain fixed.

We denote the time-dependent number of susceptible, in town and returned home agents by *S*(*t*), *I*(*t*) and *R*(*t*), respectively, and the number of workers at home and at work by *H*(*t*) and *W*(*t*), respectively. For the purpose of brevity, we will drop the explicit time dependence in our notation. The number of workers is then *N*_*w*_=*H*+*W* and the number of shoppers is *N*−*N*_*w*_=*S*+*I*+*R*. In our model, if *S*>0 and the time is between 09:00 and 18:00, arrival events of susceptible shoppers going into town, i.e. S→I, occur at a rate

$$\varepsilon +\alpha S\frac{\left(I+W\right)}{N},$$

where *ε* and *α* are positive rate constants. Thus, there is a constant underlying attraction to going into town, captured by the rate *ε*, but the overall rate at which agents go into town increases relative to the fraction of agents in town, (*I*+*W*)/*N*, but decreases as the number of susceptibles *S* decreases. Here we are using (*I*+*W*)/*N* as a proxy for whether now is a good or bad time to go into town. Departure events of agents that are in town, i.e. I→R, occur at a rate *βI*. Agents that have returned home remain in that state until 00:00 when they all become susceptible, allowing for daily periodicity.

In addition to the dynamics of shoppers, if *H*>0 and the time is between 06:00 and 10:00 then arrival events of workers going to work, i.e. H→W, occur at a rate *γ*_−_+*δ*_−_*W*. The term *δ*_−_*W* is meant to capture the morning rush hour. Similarly, if *W*>0 and the time is between 14:00 and 23:00 then departure events of agents leaving work, i.e. W→H, occur at a rate *γ*_+_+*δ*_+_*W*, where the rate *δ*_+_*W* is meant to capture the evening rush hour. These arrival and departure times are supported by a study of traffic-related air pollution [37].

The number of people arriving at a given time will be affected by their mode of transport, potentially resulting in correlations in individual arrival times. To capture this effect, we suppose that the number of shoppers that arrive (depart) during each arrival (departure) event is distributed according to some known distribution. A natural choice given the observations in §6 for this model is the (rounded) lognormal distribution whose natural logarithm has mean *μ* and variance *σ*^2^, specified as parameters.

The virtual observations, *C*(*t*), produced by our simulation correspond to the arrival or departure of each agent. Thus, if *n* agents arrive in one arrival event, the camera count is increased by *n*. These counts are cumulative but are reset to zero every hour to get the hourly counts. Importantly, the footfall counts recorded by our simulation model are free from observation error, in contrast with those recorded by the real cameras.

We implement our model using the Gillespie algorithm [16], which determines the times of each individual event. In this sense it is agent based, as we simulate the decisions made by each agent and can keep track of individual agent states over time. However, our model is relatively simple in comparison with typical ABMs and is not programmed in an object orientated framework. We dub our model the WHIRS model.

In summary, our WHIRS model state variable is *y*(*t*)=(*S*(*t*),*I*(*t*),*R*(*t*),*H*(*t*),*W*(*t*),*C*(*t*))^*T*^, the time-dependent numbers of susceptible, in town, returned home, at home and at work agents, respectively. The model runs forward in time from an initial state *y*_0_=*y*(0). The model parameters are the number of agents *N* and the number of workers *N*_*w*_=*H*+*W*; the rate parameters *ϵ*, *α* (shopper arrival), *β* (shopper departure), *γ*_±_ and *δ*_±_ (worker departure/arrival); the time periods when shoppers and workers arrive/depart. Our implementation runs for a fixed number of Gillespie steps, corresponding to individual events, or until a given end time, and outputs the model state at specified times.

## 8. Ensemble Kalman filter of the WHIRS model

In our application of the EnKF algorithm to the WHIRS model, the EnKF state vector combined the following model state variables and parameters:

$$x={\left(S(t),I(t),H(t),C(t),N,N_{w},\alpha ,\beta ,\epsilon ,\gamma ,\delta_{+},\delta_{-},\sigma ,\mu \right)}^{T}.$$

Because of the spread of magnitudes and the fact that most of the variables should be strictly positive, it proved convenient to take the natural logarithm of all but the last component (as *μ* can be negative) of the EnKF state vector *x*. We also stored all of the ensemble states so that we could easily transform back from the log variables and compute the corresponding means and covariances outside of the EnKF algorithm. We assumed that there was a 5% measurement error in the footfall count data, which we modelled as a multiplicative lognormal distribution centred on 1. This corresponds to additive Gaussian noise in log-space, as required by the linear forward model (3.2). We refer to this as the *observation error distribution*.

**Figure 5.**
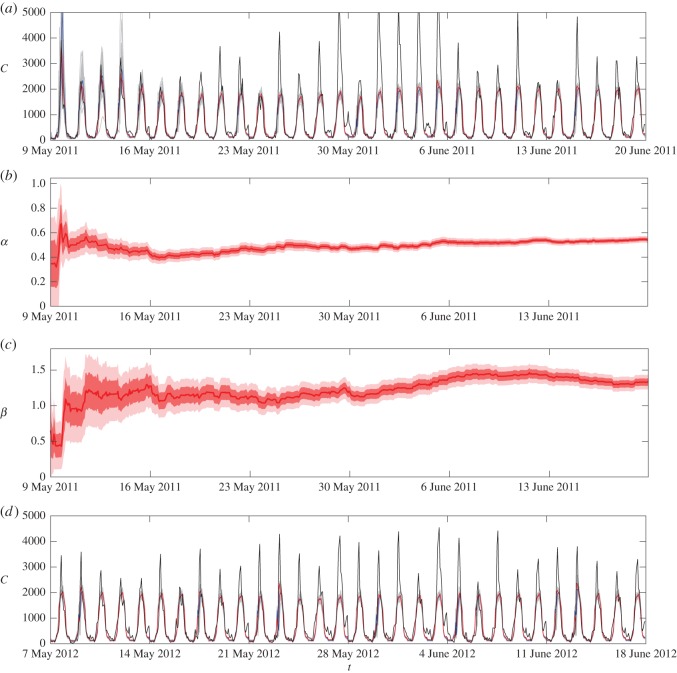
Sequential parameter estimation of the WHIRS model using Briggate footfall counts. (*a*) The hourly counts *C*(*t*_*k*_) of the EnKF forecast (blue) and analysis (red), along with the real training data (black). (*b*,*c*) The analysis for the parameters *α* and *β*, respectively. Panel (*d*) is the same format as (*a*), but for the test period data.

We chose a ‘training’ period of 30 weekdays between 00:00 on 9 May and 23:00 on 17 June 2011, over which we ran the EnKF with an ensemble size of *N*=100. Figure 5*a* illustrates the time series of footfall counts for the training period in black, the ensemble trajectories in grey, the forecasts in blue and the analysis in red. The forecast and analysis converge to a profile that gives a good representation of the daily variation away from the lunchtime peak. In figure 5*b*,*c*, we show the analysis of the rate parameters *α* and *β*, respectively. The uncertainty in the rate parameters drops considerably from the initial spread, although the values continue to change slowly. This reflects the fact that the analysis is underestimating the peak value. It is possible that we might improve on these estimates if we simulated over a longer period, but the convergence seems slow.

We now compare the EnKF forecasts from our ABM with some simple benchmark forecasts. As it takes a few days for the parameters in the EnKF to settle down, we used the final EnKF state of the training period as the initial condition for the EnKF on the testing period of 30 weekdays between 00:00 on 7 May 2012 and 23:00 on 15 June 2012. Figure 5*d* illustrates the time series of the footfall counts in black, the ensemble trajectories in grey, the forecasts in blue and the analysis in red. The RMSE between the *forecast* and the data over this period is 549.2. We also repeated the training and testing process with ensemble sizes of *N*=10 and *N*=1000, which resulted in RMSE scores of 1141 and 457.3, respectively. Thus using an ensemble size of *N*=100 produces considerably better forecasts than using *N*=10, but the gains of going to *N*=1000 are not as substantial. This demonstrates an advantage of the EnKF over particle filtering—relatively small ensemble sizes give reasonable results.

Our first benchmark forecast is the hourly mean, determined from the training data, i.e. all data prior to the testing period. Thus, the forecast for each day is exactly the same. The RMSE of this forecast over the testing period is 565.2, slightly worse than our ABM. Our other benchmark forecasts are the hour before, the same time the day before and the same time the week before. The RMSE scores for these forecasts are 519.4, 435.9 and 330.1, respectively. Given that our EnKF updates the system state each hour, we would hope that our ABM could beat the hour before forecast, as they are both using the same amount of ‘information’. The fact that the *N*=1000 ensemble does but the *N*=100 does not illustrates that the initial choice of parameters is critical. While the day before and hour before forecasts are very simple, they incorporate historical information that is not used in our ABM. Thus, these are not like-for-like comparisons and so it is perhaps not surprising that they beat our ABM forecasts. In fact, it is common with volatile data that simple forecasts can be hard to beat [38]. However, we hope that future developments of our model will allow us to improve upon these simple forecasts.

### 8.1. Rate parameter identification

In §6, we saw that the distribution of peak weekday counts is roughly lognormal and there is little correlation between peak counts on consecutive days. In §7, we described how we tried to include such daily variability in the WHIRS model, for example, by allowing multiple people to arrive at the same time. However, we can see from figure 5 that the parameters selected by the EnKF home in on a set that give rise to profiles with relatively little variability. It is likely that this is because profiles with realistic volatility may not prove to be good at forecasting. For example, if the size of the peak count is correctly predicted, but at the wrong time, the model suffers a double penalty when calculating point-wise errors [38]. In this subsection, we propose a different approach to tackle this issue that incorporates ideas from ensemble inflation methods and particle filtering [15].

As there is a weak correlation between the peak footfall counts of consecutive days, we suppose that the rate parameters, which affect the peak count, are sampled independently each day. Thus, the challenge is to determine, as soon as possible, what rate parameter best fits each day’s data. We used the training data to determine the distribution of rate parameters to sample from. Specifically, we inferred the distribution of approximate growth rates *α* by computing the gradient of the natural logarithm of the number of counts between 06:00 and 12:00 each day from the training data. This distribution was roughly normal with mean 0.5302 and s.d. 0.0851. Similarly, we inferred the distribution of approximate decay rates *β* by computing the magnitude of the gradient of the natural logarithm of the number of counts between 13:00 and 19:00 each day. This was also roughly normal with mean 0.3468 and s.d. 0.0884. The covariance between the two distributions was −0.0031.

We adapted the EnKF algorithm as follows to determine which of the growth and decay rates from the daily sample best fit the day’s data. At 00:00 of each day, we sample the rate parameters *α* and *β* from a normal distribution with mean and covariance described above. This has a similar effect to ensemble inflation, where the spread of the ensemble is artificially increased each day in order to combat the reduction in variation due to the EnKF [15]. Ensemble inflation allows the EnKF to explore the solution space to a greater extent. Instead of updating the ensemble following each hourly observation, we do so on a daily basis after making a 24 h forecast. In order to determine which values of the rate parameters are most likely each day, we use the hourly observations to determine a weighting of the ensemble, where the weight *w*_*k*_(*i*) of the *i*th ensemble member at the *k*th observation is

$$w_{k}(i)\propto w_{k-1}(i)p(z_{k}\;|\;x_{k}^{f}(i)),$$

where the constant of proportionality is chosen to normalize the weights and $p(z_{k}\;|\;x_{k}^{f}(i))$ is the observation error distribution which, as earlier, we assume to be normal for the *logarithm* of the number of footfall counts. The weights tell us each hour which ensemble members, and hence the corresponding rate parameters, are the most likely candidates for the current day’s observations. This process of updating weights is similar to a particle filter. We use an ensemble size of *N*=100 and the spread in the normalized weights typically vanishes quickly, giving a clear indication of which ensemble member matches closest to the data. This effect would be undesirable in a particle filter and is why they typically need large ensemble sizes in order to faithfully sample the posterior distribution. By contrast, the reduction in the spread of weights is advantageous here.

**Figure 6.**
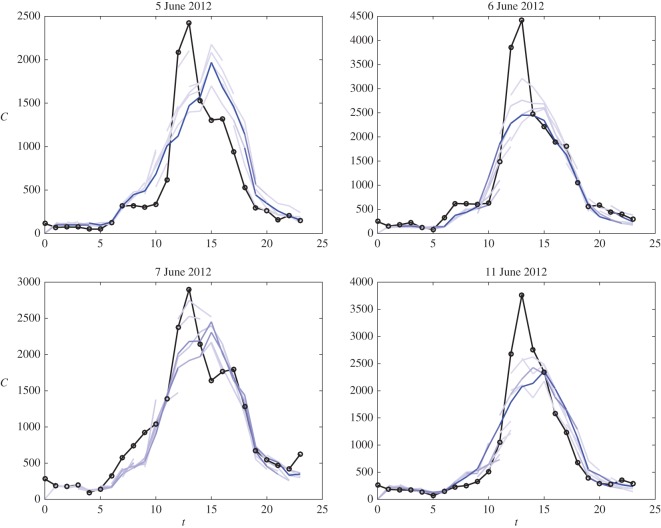
Four example days resulting from our method for estimating the rate parameters on a daily basis using a particle filter-like approach. Observation data are plotted in black and the top five hourly ensemble predictions, according to the particle filter weights, are plotted in shades of blue corresponding to the weights, blue being one and white being zero.

We ran this algorithm using the same initial conditions as those used in our EnKF described in the previous subsection. In figure 6, we illustrate the results for 4 days chosen from the 30 that we fitted. The observation data are plotted in black and the five realizations with the largest weights between each hour are plotted in blue, where the shade corresponds to the weight, with blue being a weight of one and lighter shades being less. These figures illustrate how the weights can be used to determine which ensemble member fits the observed data the best

## 9. Conclusion

In this paper, we have considered how DDA techniques, in particular the EnKF, can be applied to ABMs. Crucially, such models can provide more insight into the system state than could be achieved by standard time series or statistical methods. However, we also stress the importance of understanding the statistical properties of the data of interest in order to properly inform model development. To demonstrate the potential value of DDA to ABMs, we have investigated using the EnKF to make forecasts about footfall counts in the city of Leeds. In particular, the underlying dynamics were determined by an ABM that attempted to capture the behavioural features of people arriving in and departing from the city.

Trying to apply DDA methods to ABMs has highlighted some key challenges. As ABMs are often computationally intensive, there is a need for wider implementation of parallel computing. Also, we have not investigated here how to deal with state vectors that consist of both continuous and discrete variables. An important observation from our study is that a careful sensitivity analysis of the model parameters is also necessary in order to make accurate forecasts that faithfully reproduce real microscopic dynamics. However, ABMs often have large numbers of parameters and exploring this space and the effects these have on the dynamics is difficult manually, particularly when each realization of the model can take hours to run. This calls for numerical methods that can automatically track the boundaries between different dynamical regimes. This issue is being addressed elsewhere by so-called equation free methods [39,40], which apply numerical continuation techniques to black box models. Combining such an approach with DDA could lead to a quantitative understanding of uncertainties in ABMs and ultimately faithful models that can be used to better understand human behaviour, test scenarios of interest and make more accurate real-time predictions that can be used to reliably inform decision-making.

## Footnotes

1 Note that we assume that the map *M*_*k*_ implicitly encodes model parameters.

2 The Gillespie algorithm is an event-based method that enables efficient simulation of stochastic Poisson point processes.

## Data accessibility

The data used in this study are available from the Leeds Data Mill [13] at http://leedsdatamill.org, licensed under the terms of the Open Government Licence.
